# Efficacy of Pharmacological Interventions in Targeting Decision-Making Impairments across Substance and Behavioral Addictions

**DOI:** 10.1007/s11065-019-09400-z

**Published:** 2019-03-09

**Authors:** Samuel R. Chamberlain, Jon E. Grant

**Affiliations:** 1 Department of Psychiatry, University of Cambridge, Addenbrooke’s Hospital, Cambridge, CB2 0QQ UK; 2 Cambridge and Peterborough NHS Foundation Trust, Cambridge, UK; 3 Department of Psychiatry & Behavioral Neuroscience, Pritzker School of Medicine, University of Chicago, ISA, 5841 S. Maryland Avenue, MC 3077, Chicago, IL 60637 USA

**Keywords:** Decision-making, Gambling, Impulsivity

## Abstract

Decision-making impairments reflect tendencies towards risky or unwise choices as manifested by presence of psychiatric symptoms or cognitive impairment (e.g. representation of value, inhibitory control-response selection, learning). Such impairments are suggested by the hallmark symptoms of substance and behavioral addictions, which include escalation over time (of substance intake or a given behavior), lack of control, neglect of other domains of life, and cognitive distortions (such as ‘chasing losses’ in gambling disorder). Amongst the putative behavioral addictions, most epidemiological data exist for gambling disorder, which is now included in DSM-5 as a substance-related and addictive disorder. However, other disorders share parallels and may also constitute behavioral addictions, such as compulsive stealing (kleptomania), compulsive shopping, and compulsive sexual behavior. The current paper presents a narrative review of evidence for cognitive decision-making impairments in addictions, as well as pharmacological treatments of these disorders that may have relevance for improving decision-making. We find that objective decision-making deficits have been widely reported in patients with substance use disorders and gambling disorder, compared to controls. Decision-making in the other behavioral addictions is under-studied. Evidence-based pharmacological treatments for some of these addictive disorders, for example, opioid antagonists and glutamatergic agents, modulate neural systems playing key roles in decision-making. But clinical trials have seldom examined effects of such treatments on objective decision-making measures. Future research directions are discussed, including the need to include standardized outcome measures of decision-making (tasks and imaging) alongside traditional clinical measures, to better understand and enhance underlying treatment mechanisms.

## Introduction

Decision-making impairment can be defined, operationally, as a tendency towards risky or unwise choices as manifested by presence of psychiatric symptoms or cognitive impairment. Decision-making from a cognitive perspective is not a unitary domain but rather encompasses a number of relevant processes, including representation of value, inhibitory control, response selection, and learning (e.g. reward-outcome contingencies; Blakemore & Robbins, 2012). Impaired decision-making arising from damage to fronto-striatal pathways has long been studied by neuroscientists. Early work focused on damage to the orbitofrontal cortices leading to disinhibition, risky behavior, and personality changes (Manes et al., 2002; Rahman, Sahakia, Cardinal, Rogers, & Robbins, 2001). Of course, decision-making impairments in mental disorders do not typically arise from discrete damage, but rather from distributed (i.e. multi-regional) changes in neural networks (Clark, 2010; Guttman, Moeller, & London, 2018). These changes can conceivably arise from deviations in brain development, as well as from chronic toxic effects of psychoactive substance on these pathways, other mediators (e.g. inflammation or infection), or plastic effects of habit repetition on brain pathways (Verdejo-Garcia, Lawrence, & Clark, 2008; Yan et al., 2014). Our definitions of mental disorders are not optimal, encompassing as they do heterogeneous presentations, or even biologically ‘different’ disorders (Cuthbert & Insel, 2013). Hence there is a search for cognitive and other biologically-relevant markers that cut across relevant mental disorders, existing in a dimensional or continuous fashion in the general population, and in more extreme forms in people with mental disorders. Our premise is that the concept of decision-making may be a useful starting point in this search for such relevant markers.

Decision-making impairments are integral to understanding the clinical presentations of multiple mental disorders, especially the substance-related and behavioral addictions (Bickel et al., 2018; Koffarnus & Kaplan, 2018). It is well established that certain centrally acting drugs, such as cocaine or amphetamine, affect brain reward pathways, particularly the nucleus accumbens ‘reward centre’ and linked dopamine, glutamate, and opioid systems (Goodman, 2008; Vetulani, 2001; Volkow, Fowler, & Wang, 2004). Acute intoxication with such substances leads, clinically, to decision-making deficits, the consequences of which are readily observable in many emergency rooms on a Friday night, as well as contributing to other public health issues. For example, alcohol use predicts impulsive sexual decision-making (e.g. engaging in unprotected sex; Scott-Sheldon et al., 2016). Not only can acute intoxication lead to symptoms indicative of decision-making impairment, but also repeated consumption of such substances, for vulnerable individuals, can lead to escalating cycles of intake and functional impairment, termed addiction. Addiction encompasses a number of symptoms indicative of decision-making problems, such as (i) impaired top-down control including unsuccessful attempts to reduce intake, (ii) risky use expressed as continued, and often escalating use despite knowledge of damaging consequences, and (iii) cognitive distortions such as ‘chasing’ losses in gambling disorder, whereby an individual seeks further gambling opportunities after losing, because they perceive they are “due a pay-out”. Such symptoms are listed in the Diagnostic and Statistical Manual Version 5 for substance-related and addictive disorders (American Psychiatric Association, 2013). While research initially focused on potentially toxic effects of psychoactive substances on brain function, it is conceivable that repeated engagement in pathological behaviors could lead to plastic changes in decision-making related neural circuitry (Goodman, 1993; Grant, Brewer, & Potenza, 2006). Gambling disorder is the only currently recognized behavioral addiction in the Substance Related and Addictive Disorders DSM diagnostic category. However, several other mental disorders are characterized by repetitive engagement in rewarding habits, and have been argued to represent candidate behavioral addictions (Grant, Chamberlain, & Odlaug, 2014). For the purposes of this paper, we consider the following as behavioral addictions, in addition to gambling disorder: kleptomania (compulsive stealing), compulsive buying, and compulsive sexual behavior disorder. Substance and behavioral addictions are not new. Gambling, and its potentially untoward consequences, were discussed in ancient religious texts such as the Koran and the Talmud and ancient dice were discovered in caves dating back to 3500 BC. Excessive sexual behavior and compulsive stealing are as old as humanity. The term ‘kleptomania’ was coined around 1816 CE, due to an epidemic of young, wealthy women stealing clothes in Paris. Compulsive sexual behavior was described in the late 1700s (“nymphomania” and “satyrism” for females and males respectively), while compulsive shopping (previously termed “oniomania”) dates back to nineteenth century America or earlier.

This review paper seeks to address two hypotheses: (i) that substance and behavioral addictions, as defined as above, are characterized by objective decision-making deficits on cognitive tasks dependent on fronto-striatal circuitry, and (ii) that pharmacological treatments with efficacy in these disorders may act partly via modulation of fronto-striatal circuitry and neurochemical systems crucially involved in decision-making.

## Methods

Literature searches were conducted in PubMed for data papers and meta-analyses addressing (i) cognitive findings in substance use disorders and behavioral addictions compared to controls (scope of disorders defined per the introduction), (ii) pharmacological treatment studies in substance use disorders and behavioral addictions. Due to the thematic breadth of the paper and variability in the decision-making measures utilized across the literature, we opted pragmatically for a narrative selective review rather than a systematic review or meta-analysis.

## Results

### Decision-Making in Substance Use Disorders

**Fig. 1:**
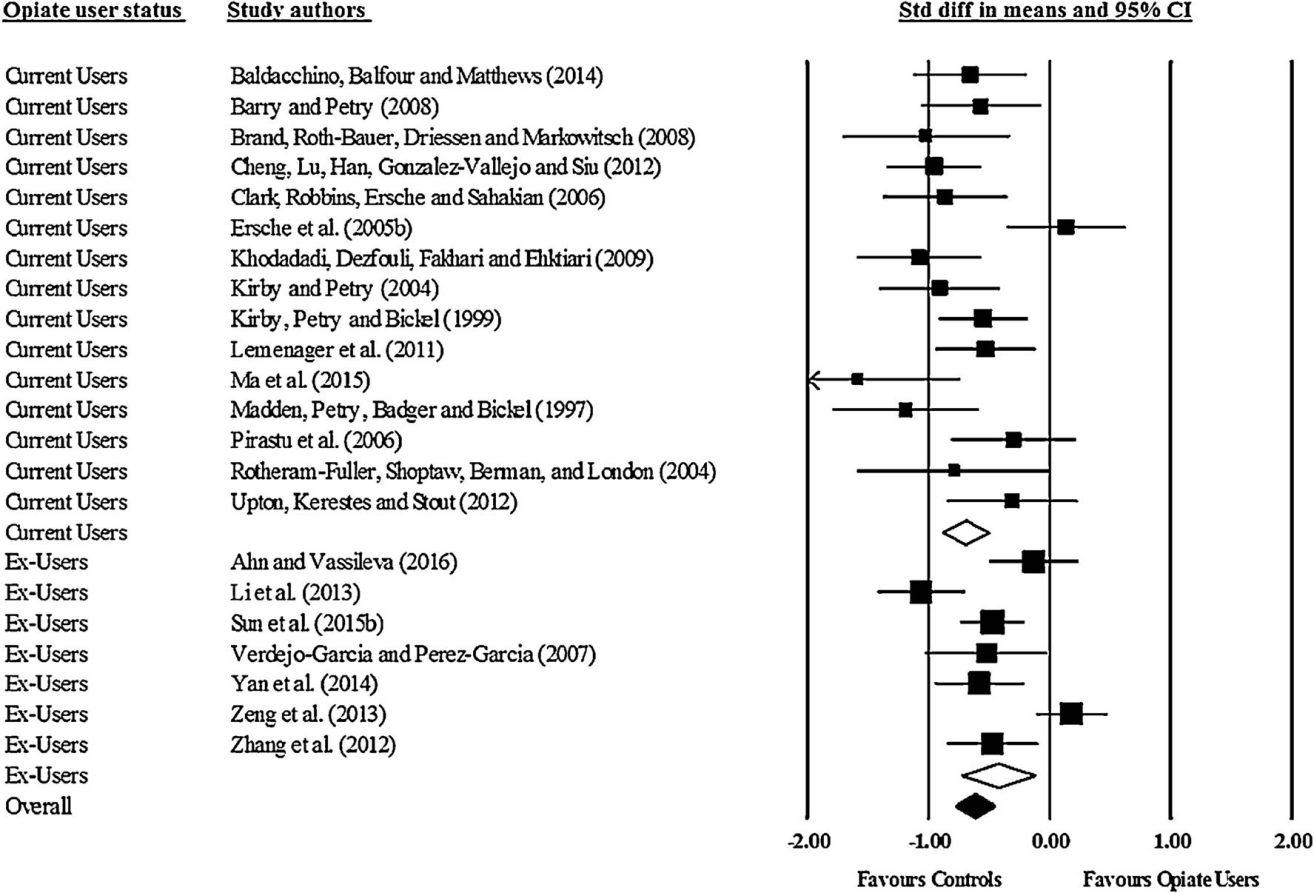
Meta-analysis of decision-making performance on cognitive tasks, between substance users and controls. Taken from and reprinted with permission from Biernacki et al, 2016. Copyright Elsevier 2016

We found that decision-making functions had been widely examined in case-control studies for several of the substance use disorders, particularly for alcohol use and opiate use disorders. We thus focus specifically on these substance use disorders. In a meta-analysis of approximately 15 studies (Biernacki, McLennan, Terrett, Labuschagne, & Rendell, 2016), significant decision-making impairment was found in current opioid users versus controls, with a medium effect size (Fig. 1; overall d = 0.51). Interestingly, decision-making impairment was also significant versus controls for ex-drug users (~ 7 studies), and appeared similar to that observed in studies of current patients (Fig. 1). There was considerable variability in the decision-making tasks and measures used across the data studies included in this meta-analysis. The studies had moderate heterogeneity and the overall fail-safe N was 38, indicating that 38 additional ‘negative’ studies would be needed to nullify the significant effect. The most commonly used task quantifying decision-making in these data studies was the Iowa Gambling Task, but studies also used Delay Discounting task, Cambridge Gambling Task, Information Sampling Task, Balloon Analogue risk task, and the Soochow Gambling task (Biernacki et al, 2016). In another meta-analysis, decision-making deficits were found on the Iowa Gambling Task in alcohol use disorder patients (*N* = 500) compared to controls, with medium effect size (d = 0.581; Kovacs, Richman, Janka, Maraz, & Ando, 2017). There was no evidence of publication bias based on plot inspections, but there was moderate heterogeneity across studies.

In a meta-analysis focusing on discounting, cutting across the substance use literature (including nicotine, cocaine, stimulants, heroin, opiates, cannabis, but also gambling), a total of 64 data papers were identified (most data were available for alcohol and tobacco use; MacKillop et al., 2011). Across all studies, addiction was associated with significantly higher reward discounting, indicating relative decision-making deficits when compared to controls, with small effect size (d = 0.15), but with high heterogeneity. The overall effect size was in fact medium-large for the Kirby questionnaire (d = 0.63) indicating that some measures were more sensitive to effects of substance addiction than others. In a more recent meta-analysis, again a range of addictions were considered together for discounting tasks, but this time with a focus on continuous-dimensional measures of substance use (*N* = 138 effect sizes pooled; Amlung, Vedelago, Acker, Balodis, & MacKillop, 2017). Addictive behavior was significantly associated with increased delay discounting with small effect size (r = 0.14), with substantial heterogeneity across studies. There was low evidence for publication bias.

### Decision-Making in Gambling Disorder

Several meta-analyses were identified that explored decision-making in people with gambling disorder compared to controls. In the earlier described meta-analysis by Kovacs and colleagues, gambling disorder literature was also considered as well as alcohol use disorder (Kovacs, Richman, Janka, Maraz, & Ando, 2017). The authors reported significantly impaired Iowa Gambling Task performance in gambling disorder (*N* = 292) versus controls, with large effect size (d = 1.034). Interestingly this impairment was statistically larger than that found in the alcohol use disorder literature. There was no evidence for publication bias based on funnel plots, but there was high heterogeneity. In the meta-analysis of MacKillop and colleagues (MacKillop et al., 2011), seven studies were found examining discounting in gambling disorder versus controls. Gambling disorder was associated with significantly increased discounting (i.e. worse decision-making) compared to controls, with medium effect size (d = 0.79), and these studies appeared to be more homogenous than those for other behaviors that were considered such as alcohol and tobacco. Another meta-analysis focused on discounting in gambling, and reported that gambling was associated with shallower probability discounting than controls (*n* = 12 samples; overall g = 0.36; Kyonka & Schutte, 2018). There was no evidence of publication bias, and there was low heterogeneity. Shallower discounting implied that gamblers assigned relatively more value to low probability gains, or high probability losses, than would be appropriate (Kyonka & Schutte, 2018). In a meta-analysis of compulsivity-related cognitive performance in gambling disorder, which included some tasks germane to decision-making, gambling disorder was associated with significant impairment in Wisconsin Card Sorting task performance (*N* = 9 studies; d = 0.518, medium effect size) and Set-Shifting task performance (*N* = 3 studies; d = 0.412, medium effect size; van Timmeren, Daams, van Holst, & Goudriaan, 2018). Heterogeneity was low and assessment of publication bias was not tenable due to the relatively small number of studies in the published literature.

### Decision-Making and Other Behavioral Addictions

Substance use disorders and gambling disorder aside, our literature search yielded sparse cognitive data for the other behavioral addictions in regard to decision-making. One study explored cognition in a group of people who had shoplifted over the preceding year, and found decision-making impairment on the Cambridge Gambling Task (Grant, Chamberlain, & Odlaug, 2012, 2014). Shoplifters appeared relatively intact on the other neurocognitive domains that were examined. Another study indicated that individuals with compulsive buying had impairments in risk adjustment during decision making (Cambridge Gambling Task) (Derbyshire, Chamberlain, Odlaug, Schreiber, & Grant, 2014). In a relatively small study of people with compulsive sexual disorder, no significant cognitive differences were found on the Cambridge Gambling Task, nor other tasks that were included (Derbyshire & Grant, 2015). Due to paucity of data, firm conclusions cannot be drawn about decision-making deficits in these candidate disorders.

### Potential Neurobiological Underpinnings of Decision-Making Abnormalities in Substance and Gambling Disorders

Compared to controls, extensive literature has identified structural and functional brain differences in people with substance and gambling addictions. For example, in a meta-analysis of structural brain changes associated with stimulant use disorders, reductions of prefrontal cortical region grey matter were observed (Ersche, Williams, Robbins, & Bullmore, 2013). Decreased thickness of frontal grey matter has been observed in gambling disorder, even up to 20% reductions in cortical thickness (on average) in treatment resistant gambling disorder cases compared to controls (Grant, Odlaug, & Chamberlain, 2015). Another separate study also reported grey matter reductions in the frontal cortex in gambling disorder, and as with the Grant et al. study, comorbid substance use disorders were tightly controlled for (Zois et al., 2017). The grey matter changes extended to a wider range of cortical regions when comorbid cases (gambling and substance addiction) were considered.

A recent meta-analysis of functional neuroimaging studies across a range of substance and behavioral addictions was conducted (data from 25 studies: Luijten, Schellekens, Kuhn, Machielse, & Sescousse, 2017). People with gambling addiction showed hypoactivation of the striatum, as did people with substance addiction, during reward anticipation, compared to controls. During reward outcomes, substance addiction was associated with hyperactivation of the ventral striatum, whereas gambling addiction was associated with hypoactivation of the dorsal striatum. Thus, depending on the task process being considered, these two types of disorders show similar or opposite functional abnormalities in sub-cortical regions during decision-making (Luijten, Schellekens, Kuhn, Machielse, & Sescousse, 2017). Some differences were also observed in cortical regions, though the paper focused on sub-cortical findings due to the emphasis on reward-processing. The different abnormalities observed in gambling disorder during reward outcomes (hypoactivation of dorsal striatum) compared to substance use disorders (hyperactivation of ventral striatum) may in our view reflect either the chronic effects of substance use on reward-related brain pathways in substance use disorders, or different stages of disease in the shift from impulsive reward-driven behavior (ventral striatum) to compulsive habitual behavior (dorsal striatum).

These imaging data are consistent with the idea that decision-making abnormalities in substance use and gambling disorders may reflect structural or functional abnormalities of frontal cortical regions, as well as the ventral and dorsal striatum. We found a severe paucity of imaging studies for the other candidate behavioral addictions.

## Evidence-Based Pharmacological Treatments for Addictions

Pharmacological treatment options for substance use disorders exist based on reasonably strong clinical trial evidence. By contrast, the evidence-base for treating behavioral addictions is partial, characterized by the existence of only a handful of rigorously conducted high quality trials. Our literature search found that very few clinical trials included objective decision-making measures, hence this section is mostly restricted to examining effects on symptoms per se.

### Substance Use Disorders

Examination of controlled trial data for the broad spread of substance use disorders is outside the scope of the current paper due to the volume of literature. Readers are referred to existing reviews such as (Reus et al., 2018; van den Brink, 2012: see also http://cda.cochrane.org/our-reviews). Here, we focus on treatment data for alcohol use disorder as an example, which is common and often treated in clinical practice, and for which a fair body of controlled trial data exist. Some data exist for the use of various medications in alcohol use disorder (e.g. SSRIs, topiramate, baclofen), which are discussed in excellent reviews elsewhere (Akbar, Egli, Cho, Song, & Noronha, 2018; Holt & Tobin, 2018; Kim, Hack, Ahn, & Kim, 2018). We focus subsequentially here on alcohol use disorder treatments formally approved in at least some jurisdictions. In a systematic review and meta-analysis of drug treatments for alcohol use disorder in outpatient settings, sufficient controlled trial data were available to examine acamprosate, disulfiram, and naltrexone (Jonas et al., 2014). These medications were used in addition to psychosocial support. Acamprosate has complex and disputed mechanisms of action including possible effects on the GABAergic and N-methyl-D-aspartate (NMDA) receptor systems (Littleton & Zieglgansberger, 2003). Compared to placebo treatment, acamprosate was associated with significant reductions in the likelihood of returning to drinking over time. Disulfiram (inhibitor of acetaldehyde dehydrogenase) did not discriminate significantly from placebo on this measure. For the opioid antagonist naltrexone, benefits were observed for reduction in return to drinking compared to placebo, but mainly at one particular dose (50 mg/day orally). For a more detailed overview of the trial data, the reader is referred to (Jonas et al., 2014).

### Gambling Disorder

Different types of pharmacological agents have been investigated in the management of gambling disorder, including serotonin reuptake inhibitors (SRIs), opioid antagonists, glutamatergic agents, and the anti-dopaminergic medication olanzapine. One trial used the SRI sertraline, and found ~70% treatment response irrespective of whether patients received active treatment or placebo, that is, this was a robust negative result (Saiz-Ruiz et al., 2005). Two studies examined the SRI fluvoxamine and two studies examined paroxetine. For each of these two medications, one trial identified significant benefit versus placebo, and one trial was negative overall (Blanco, Petkova, Ibanez, & Saiz-Ruiz, 2002; Grant et al., 2003; Hollander et al., 2000; Kim, Grant, Adson, Shin, & Zaninelli, 2002). As such, the overall body of evidence for SRIs in gambling disorder is mixed. It may be that some patients are more likely to respond to SRIs, such as in the presence of comorbid major depressive disorder or obsessive-compulsive disorder.

Positive treatment data have been reported using opioid antagonists in gambling disorder. Naltrexone, an antagonist at mu opioid receptors, and to a lesser extent sigma opioid receptors, with efficacy in alcohol use disorder (see above), showed significant symptomatic benefit over placebo for gambling disorder in two trials (Grant, Kim, & Hartman, 2008; Kim, Grant, Adson, & Shin, 2001). Nalmefene, an antagonist at the mu opioid receptor, showed significant benefit over placebo in two trials, albeit one of these trials did not yield a significant benefit when a more conservative intent-to-treat approach was used (Grant, Odlaug, Potenza, Hollander, & Kim, 2010; Grant et al., 2006).

Another promising area for the treatment of gambling disorder is the use of glutamate modulating agents. N-acetylcysteine (NAC) is an amino acid prodrug that dampens glutamatergic transmission in preclinical models of addiction (Kalivas, Lalumiere, Knackstedt, & Shen, 2009). In one human trial, NAC treatment showed clear efficacy over placebo in gambling disorder (83.3% still met responder criteria at the end of the double-blind phase, compared with only 28.6% of those assigned to placebo: Grant, Kim, & Odlaug, 2007). Another study was run using NAC in nicotine-dependent pathological gamblers, on the basis that patients with a “double whammy” of both substance and behavior addiction might benefit from it. This study found that initial treatment with NAC and psychotherapy was associated with lower gambling symptoms some months later (after treatment had ended), compared to initial treatment with placebo and psychotherapy (Grant et al., 2014b). Another candidate glutamateric agent is topiramate, though this medication has a problematic side effect profile and has important neurochemical actions at other sites besides the glutamate system. In one study, topiramate did not differentiate from placebo in the treatment of gambling disorder (Berlin et al., 2013). Lastly, for gambling disorder, despite dopamine being implicated in its pathophysiology, two trials found that the dopamine antagonist olanzapine was no more effective than placebo (Fong, Kalechstein, Bernhard, Rosenthal, & Rugle, 2008; McElroy, Nelson, Welge, Kaehler, & Keck, 2008). These negative results may reflect dissociable roles for dopamine in different brain regions and it is conceivable that pro-dopaminergic agents selectively acting on the cortex may have utility, as discussed further in a later section.

### Other Candidate Behavioral Addictions

For behavioral addictions besides gambling disorder, even fewer controlled, double-blind clinical trials exist. For kleptomania, one study randomized participants to the SRI escitalopram or placebo (double-blind) after initial treatment with open-label escitalopram. Relapse rates did not differ significantly between the treatment arms indicating that escitalopram did not differentiate from placebo (Koran, Aboujaoude, & Gamel, 2007). One trial in kleptomania found that the opioid antagonist naltrexone was superior to placebo given over 8 weeks (Grant, Kim, & Odlaug, 2009). In an open-label study, 8-week treatment with memantine was associated with significant improvements in symptom severity and reductions in cognitive impulsivity (Grant, Odlaug, Schreiber, et al., 2013). For compulsive buying disorder, all double-blind placebo-controlled trials used SRIs. Two trials reported that fluovoxamine was not superior to placebo in the treatment of compulsive buying (Black, Gabel, Hansen, & Schlosser, 2000; Ninan et al., 2000). In a study that used open-label active treatment followed by double-blind discontinuation, active treatment did not discriminate from placebo continuation, indicating a negative result Koran, Aboujaoude, Solvason, et al., 2007). In a 10-week open-label study using memantine in compulsive buying, hours and money spent on shopping reduced significantly, and cognitive tasks of impulsivity improved significantly (Grant, Odlaug, Mooney, O'Brien, & Kim, 2012). For compulsive sexual behavior, the only published controlled trial found that the SRI citalopram reduced some aspects of sexual desire (desire for sex, frequency of masturbation, and frequency of pornography use), versus placebo (Wainberg et al., 2006). However, no overall benefits on risky behaviors were detected compared to placebo. Some patients may display paradoxical increases in risky behavior to compensate from reduced sexual drive due to SRI treatment.

## New Pharmacological Directions Based on the Neurosciences

Studies have found that substance-dependent individuals have a blunted dopaminergic response to amphetamine or methylphenidate challenge, which would ordinarily increase extra-cellular dopamine levels by blocking reuptake and triggering release (Del Campo, Chamberlain, Sahakian, & Robbins, 2011). Sufficient research has been conducted in the context of stimulant-dependence to conduct a meta-analysis. Ashok and colleagues examined a total of 31 studies and found a significant blunting of striatal dopamine release in stimulant-dependent patients compared to controls with a medium-large effect size (Ashok, Mizuno, Volkow, & Howes, 2017). There was also a significant decrease in dopamine D2/D3 receptor availability with medium-large effect size. Radioligand data suggest that patients with gambling disorder – especially at the more severe end – may have an over-active striatal dopaminergic system, which is in contrast with findings in substance dependence, which may be characterized by a blunted dopaminergic system (Boileau, Payer, Chugani, Lobo, Behzadi, et al, 2013; Boileau, Payer, Chugani, Lobo, Houle, et al, 2014). However, to our knowledge, direct head-to-head radioligand comparisons of gambling disorder and substance use disorders are lacking, as are radioligand data for other behavioral addictions.

These radioligand data lead to the logical question: could dopaminergic medication worsen or improve addictions, via effects on striatal dopamine? Indeed, pro-dopaminergic medication has been linked with apparent new onset, or worsening of, behavioral addictions (e.g. shopping, sex, gambling) in some cases of Parkinson’s Disease (Ambermoon, Carter, Hall, Dissanayaka, & O'Sullivan, 2011; Bugalho & Oliveira-Maia, 2013; Poletti et al., 2013) and recently by a small number of people taking aripiprazole (Roxanas, 2010). Interestingly, there is some evidence supporting the efficacy of aripiprazole in the treatment of alcohol dependence (Anton et al., 2008; Martinotti, Di Nicola, Di Giannantonio, & Janiri, 2009). Thus the direction of effect may differ depending on baseline dopamine function and the presence of particular disorder(s). Only some individuals have such adverse events, perhaps those with a stronger familial history of addictions. Theoretically, at least some cases of behavioral addiction could be mediated by excess sub-cortical dopamine drive, which may be expected to be improved by antipsychotic (dopamine antagonist) medication. As discussed above, findings to date with such medications in substance and behavioral addictions have been disappointing. It has been suggested that aripiprazole (dopamine antagonist), or modafinil (wake promoting agent with indirect dopaminergic effects) could dampen alcohol-seeking behavior and promote abstinence, but controlled trials are lacking (Martinotti et al., 2016; Zack & Poulos, 2009). In the latter study, modafinil reduced desire to gamble in high impulsive subjects, and had the opposite effect in low impulsive subjects. Thus, again, effects of a medication are likely to be contingent on baseline status.

In conditions of low baseline dopamine, it is conceivable that pro-dopaminergic medications such as psychostimulants could enhance aspects of decision-making. In children with attention-deficit hyperactivity disorder (ADHD), acute methylphenidate led to more conservative decision-making on the Cambridge Gambling Task, compared to placebo (DeVito et al., 2008). Some positive effects have also been reported with acute methylphenidate in elderly patients with fronto-temporal dementia (Rahman et al., 2006) and when using a selective norepinephrine reuptake inhibitor in patients with Parkinson’s Disease (Kehagia et al., 2014). Cambridge Gambling Task performance has also been reported to be affected by putative dopamine depletion in healthy volunteers, and in people with a history of depression (McLean, Rubinsztein, Robbins, & Sahakian, 2004; Roiser et al., 2005). Clinical trials of psychostimulant or dopamine or norepinephrine reuptake inhibition medications in substance and behavioral addictions may be of future research interest.

An alternative therapeutic proposition would be to selectively enhance cortical dopamine, thereby ameliorating decision-making dysfunction. Positive open-label data have been reported in the context of gambling disorder using tolcapone (Grant, Odlaug, Chamberlain, et al., 2013). Tolcapone inhibits the main enzyme responsible for degradation of cortical dopamine and was found to exert its beneficial effects on gambling, at least partly, via enhancement of decision-making related frontal circuitry. In separate work, gamblers with the ‘val/val’ genetic variant of this same enzyme showed worse decision-making on the Cambridge Gambling Task, suggesting that medications capable of blocking this enzyme may be particularly useful in individuals with low baseline levels of cortical dopamine (Grant, Odlaug, Chamberlain, et al., 2013). This is the case since the ‘val/val’ genetic variant is associated with much higher enzyme activity, and thereby faster breakdown on extra-synaptic dopamine.

## Conclusions

A recent shift in psychiatry and the neurosciences has been towards trying to decompose top-level phenotypes (symptoms) into intermediate biological markers (Cuthbert & Insel, 2013). Such measures may then, conceivably, act as intermediaries to better understand the relationships between genetic-environmental risk factors and the ultimate expression of psychiatric syndromes (Chamberlain, Stochl, Redden, & Grant, 2017). Neuropsychological tests relating to decision-making are likely to be relevant in this search, given that decision-making problems are suggested by the symptoms of substance and behavioral addictions and this premise formed the basis for the current narrative review. Our first hypothesis, namely that addictions are associated with decision-making impairments on cognitive tasks, was partly supported by our literature search. We found meta-analytic support, from the literature, for the existence of decision-making impairments in several types of substance use disorders (e.g. alcohol, opioid), and in gambling disorder. The magnitude of effect appeared larger and with lower heterogeneity in relation to gambling disorder. Evidence of decision-making deficits in other behavioral addictions (kleptomania, compulsive shopping, compulsive sexual behavior disorder) was partial only, with a poverty of data studies, highlighting the need for much more research into these neglected areas of mental health. Rather than refuting our hypothesis, rather our findings inform the need for more research in these settings. Furthermore, neurochemical systems and fronto-striatal circuitry underpinning decision-making (Clark et al., 2008; Clark, Cools, & Robbins, 2004; Fellows & Farah, 2005; Preuschoff, Bossaerts, & Quartz, 2006; Simon et al., 2011; Talbot, Watson, Barrett, & Cooper, 2006) are implicated in the pathophysiology of addictive disorders (Clark & Limbrick-Oldfield, 2013; de Ruiter, Oosterlaan, Veltman, van den Brink, & Goudriaan, 2012; Goudriaan, Yucel, & van Holst, 2014; Rogers et al., 1999; Verdejo-Garcia, Chong, Stout, Yucel, & London, 2017). Thus, based on the evidence so far, these decision-making impairments observable in substance and gambling addictions may be linked with reductions in cortical grey matter (Ersche, Williams, Robbins, & Bullmore, 2013; Grant, Odlaug, & Chamberlain, 2015) coupled with functional abnormalities of the frontal cortices and basal ganglia (ventral and dorsal striatum: Luijten, Schellekens, Kuhn, Machielse, & Sescousse, 2017). Regions such as these are richly modulated by different neurochemical systems, which may provide a new vista on future treatment directions as well as helping to account for positive data for some glutamatergic agents and opioid antagonists.

Our second hypothesis, that treatments with efficacy in these disorders may act via modulation of fronto-striatal circuitry involved in decision-making was not directly addressable due to an absence of relevant data. Medications with the firmest evidence for treating addictions include those acting on the glutamatergic and opioid systems, which are known to play key roles in decision-making and fronto-striatal circuitry. However, few clinical trials in addiction had included objective cognitive measures of decision-making. There were some open-label pilot data suggesting that memantine may improve decision-making (impulsivity) in compulsive shopping and kleptomania (Grant et al., 2012; Grant, Odlaug, Schreiber, et al., 2013). To address this second hypothesis further would require future clinical trials for addictions that include not only traditional clinical outcome measures but also cognitive outcome measures.

It is seen from this selective overview of the existing addiction literature that while pharmacological treatments exist for the symptomatic treatment of addictions linked with decision-making impairments, it is not yet clearly established that objective improvements in decision-making on laboratory-based tasks are directly linked with symptomatic improvement. Because cognitive impairments germane to decision-making appear common in addictive disorders, it would be valuable for future clinical trials to include such measures alongside the more traditional symptom outcome measures (Goodie & Fortune, 2013; Grant, Chamberlain, Schreiber, Odlaug, & Kim, 2011). The current review enables recommendations to be drawn together for such future work, including the need to minimize heterogeneity by using highly validated reliable cognitive tasks, the need to take a translational approach in clinical trials by including imaging and cognitive measures, and the need for more research into behavioral addictions in general, several of which are extremely neglected from clinical and research perspectives. In addition to examining pharmacological and psychological treatments for decision-making deficits, brain modulatory techniques should also be investigated.
